# Theoretical Insights into the Solvent Polarity Effect on the Quality of Self-Assembled *N*-Octadecanethiol Monolayers on Cu (111) Surfaces

**DOI:** 10.3390/molecules23040733

**Published:** 2018-03-22

**Authors:** Jun Hu, Shijun He, Yaozhong Zhang, Haixia Ma, Xiaoli Zhang, Zhong Chen

**Affiliations:** 1 School of Chemical Engineering, Northwest University, Xi’an 710069, Shaanxi, China; heshijun0717@163.com (S.H.); mahx@nwu.edu.cn (H.M.); xlzhang@nwu.edu.cn (X.Z.); 2 State Key Laboratory of Eco-Hydraulics in Northwest Arid Region of China, Xi’an University of Technology, Xi’an 710048, Shaanxi, China; zhangyz@xaut.edu.cn; 3 School of Materials Science and Engineering, Nanyang Technological University, 50 Nanyang Avenue, Singapore 639798, Singapore

**Keywords:** copper, corrosion, density functional theory, solvent polarity, self-assembled monolayer

## Abstract

The effect of solvent polarity on the quality of self-assembled *n*-octadecanethiol (C_18_SH) on Cu surfaces was systematically analyzed using first-principles calculations. The results indicate that the adsorption energy for C_18_SH on a Cu surface is −3.37 eV, which is higher than the adsorption energies of the solvent molecules. The higher adsorption energy of dissociated C_18_SH makes the monolayer self-assembly easier on a Cu (111) surface through competitive adsorption. Furthermore, the adsorption energy per unit area for C_18_SH decreases from −3.24 eV·Å^−2^ to −3.37 eV·Å^−2^ in solvents with an increased dielectric constant of 1 to 78.54. Detailed energy analysis reveals that the electrostatic energy gradually increases, while the kinetic energy decreases with increasing dielectric constant. The increased electrostatic energies are mainly attributable to the disappearance of electrostatic interactions on the sulfur end of C_18_SH. The decreased kinetic energy is mainly due to the generated push force in the polar solvent, which limits the mobility of C_18_SH. A molecular dynamics simulation also confirms that the -CH_3_ site has a great interaction with CH_3_(CH_2_)_4_CH_3_ molecules and a weak interaction with CH_3_CH_2_OH molecules. The different types of interactions help to explain why the surface coverage of C_18_SH on Cu in a high-polarity ethanol solution is significantly larger than that in a low-polarity *n*-hexane solution at the stabilized stage.

## 1. Introduction

Copper (Cu) and its alloys have been widely used in many industrial sectors, including electronic, chemical, and ocean engineering [1]. Despite its many outstanding properties, Cu is very chemically active, and is thus prone to corrosion. Serious corrosion not only leads to grave economic loss, it also poses a potential threat to human life [2,3,4]. Various ways to protect metals from corrosion have been developed based on different principles. Self-Assembled Monolayers (SAMs) are one of the most economic, highly efficient, and simple ways to protect metals and alloys from corrosion and oxidization [5,6,7]. Experimental research has been carried out to identify the protection mechanism of SAMs on Cu surfaces. It was generally believed that the densely packed monolayers were formed through chemisorption onto the surface of Cu [8]. Since the properties of the solvent affect the assembly of SAMs, the qualities of the SAMs formed in different solvents are expected to be different. Thus far, many researchers have studied the thiol-SAMs formed on gold surfaces in different solvents. Among them, Bain et al. evaluated the effect of various solvents (dimethylformamide, tetrahydropyranyl, ethanol, carbon tetrachloride, acetonitrile, hexadecane, cyclooctane, and toluene) on the formation of SAMs on gold surfaces. It was found that the hexadecanethiol monolayer adsorbed on gold in a hexadecane solution displays a low contact angle when it reaches certain thickness, which can possibly be attributed to the incorporation of hexadecane into the monolayer [9]. Dai et al. have reported the effects of solvents on the quality of the SAM of dodecanethiol on gold. They revealed that the solvent parameters (such as polarity, solubility, molecular size, octanol-water partition coefficients, and viscosity) affect the quality of the C_12_SH SAMs [10]. Ujjal et al. have also proposed that the nature of the solvent might affect the blocking properties and barrier characteristics of the –CH_3_ terminated SAMs [11,12]. Our previous experiments by electrochemical impedance spectroscopy, Fourier transform infrared spectroscopy, and X-ray photoelectron spectroscopy, showed that the qualities of self-assembled *n*-octadecanethiol (C_18_SH) indeed differ when it is formed in different solvents (*n*-hexane, toluene, trichloroethylene, chloroform, acetone, acetonitrile, and ethanol) [13]. Based on the experimental results, the assembly is likely to be a competitive adsorption process, containing interactions among the solvent, the solute, and the surface. However, which of these interactions plays an important role during the self-assembly process remains unclear due to the potential complexity of interactions among the different entities.

Computational analysis, on the other hand, is able to overcome the experimental limitations, and thus provides a very useful tool to understand the assembly mechanisms [14,15,16,17]. Benchouk et al. studied the effect of solvents on the 1.3-dipolar cycloaddition of benzonitrile *N*-oxide with cyclopentene using first-principles calculations. They found that solvent polarity leads to the slow inhibition of the 1,3-dipolar cycloaddition due to the low polarity of the transition state [18]. Sainudeen et al. analyzed the solvent polarity of zwitterionic merocyanine using quantum chemical calculations. They found that solvents play a remarkable role in the structure and in the first hyperpolarizability of merocyanine monomers and aggregates [19]. Thus far, these attempts to elucidate the adsorption process remain at a molecular level, and most of them were carried out in vacuum or water solution conditions.

In this paper, a comprehensive analysis of the effect of solvent polarity is presented from the perspective of interactions among the solvent, the solute, and the surface. First, the electronic structure of C_18_SH, C_18_S, and different solvents are considered. Then, the adsorption of different solvent molecules on the Cu (111) surface is calculated to interpret the interaction between the solvent and the surface. Based on the simulation, the effect of solvent polarity on the quality of C_18_SH SAMs on the Cu surface is explained through the proposed mechanisms. This work helps us to better understand the micro-mechanisms of solvent polarity effects of C_18_SH on pure Cu surfaces. The method can be extended to understand interactions between other SAMs and metal surfaces.

## 2. Results

### 2.1. Electronic Structure of C_18_SH, C_18_SH and Different Solvents

**Figure 1:**
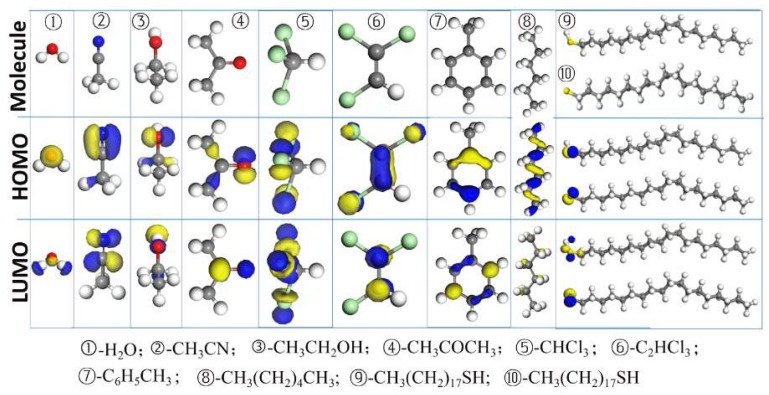
The map of the HOMO and LUMO for different molecules with an isovalue of ±0.10 e.

The maps of the Highest Occupied Molecular Orbital (HOMO) and the Lowest Unoccupied Molecular Orbital (LUMO) are shown in Figure 1. The quantitative results of quantum chemical parameters are as listed in Table 1.

The HOMO and LUMO regions of different molecules are mainly contributed by O, N, and S elements and the benzene ring. This indicates that these are the adsorption sites on the metal surface. As indicated in Table 1, the energy of LUMO, *E_L_*, is greatly reduced after C_18_SH dissociates into C_18_S. *E_L_* represents the electron acceptability, which is directly related to the electron affinity and characterizes the susceptibility of the molecule against attacks by nucleophiles. The lower value of *E_L_* means stronger electron acceptability of the molecules, indicating the strong interaction between the Cu surface and C_18_S. This is also verified through the increased fraction of electron transfer, Δ*N,* from the Cu surface to C_18_S. Furthermore, it is noted that the difference in Δ*N* between C_18_SH and different solvent molecules is not very high.

### 2.2. The Adsorption for C_18_SH, C_18_S and Solvent Molecules on Cu Surface

**Figure 2:**
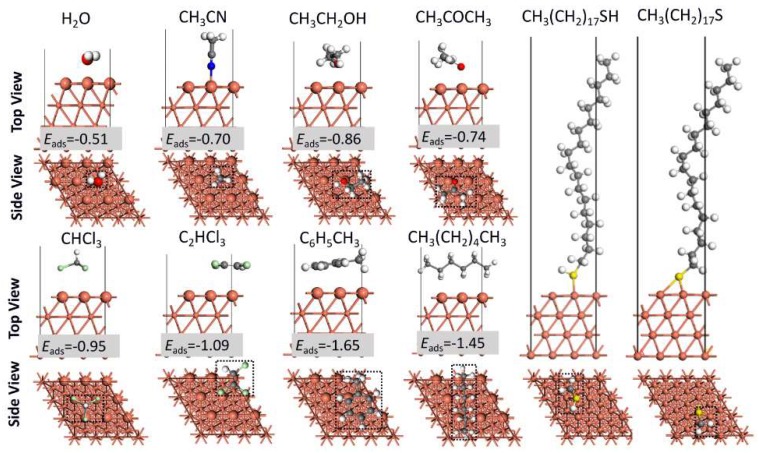
The adsorption structures of different solvent molecules in corresponding solvents as well as C_18_SH and C_18_S in a water solution. The unit of adsorption energy is in eV. More details are given in Table S1.

**Figure 3:**
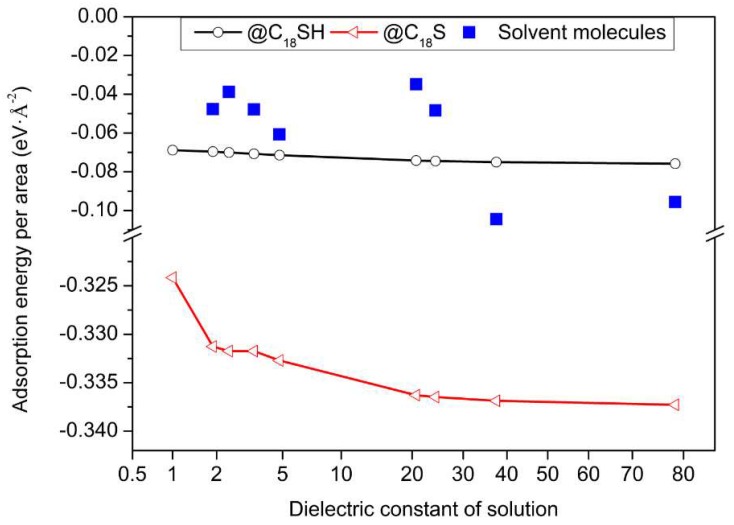
The adsorption energies of C_18_SH, C_18_S, and different solvent molecules in different solvents. The x-axis labelled for the dielectric constant of 1-Vacuum; 1.89–CH_3_(CH_2_)_4_CH_3_; 2.40–C_6_H_5_CH_3_; 3.42–C_2_HCl_3_; 4.81–CH_3_Cl_3_; 20.7–CH_3_COCH_3_; 24.3–CH_3_CH_2_OH; 37.5–CH_3_CN; 78.5–H_2_O.

The stable adsorption structures and energies of C_18_SH and C_18_S (the dissociated state of C_18_SH) on the Cu (111) surface in different solvents are shown in Figure 2 and Figure 3, where the stable adsorption energies of solvent molecules are also added and compared. The dominant effect is due to interactions between sulfur group and the Cu surface, based on the well-known hard-soft concept of Pearson.

As shown in Figure 2, H_2_O adsorbs preferentially at the top site, with its molecular plane parallel to the surface. This result is consistent with a previous STM observation, which indicates an atop adsorption site [20]. Furthermore, the adsorption energy for water on a Cu surface is −0.51 eV, which is also consistent with the reported value of 0.51–0.55 eV [21], 0.54–0.57 eV [22], and 0.42 eV [23]. For the solvent molecules, elements O, N, S, and Cl are easily absorbed by the Cu atoms, because these atoms have many electrons and prefer the acidic Cu sites. Furthermore, it can be seen that only CH_3_CN is vertically adsorbed to the surface; others molecules are parallel to the surface. The covered area (the dotted black line in Figure 2) for C_6_H_5_CH_3_ and CH_3_(CH_2_)_4_CH_3_ is larger, due to their bigger molecule sizes. In order to obtain the strength of interaction between the Cu surface and different solvent molecules, the adsorption energy per unit area is plotted and compared, as shown in Figure 3. The adsorption energies are not much different for most solvent molecules and C_18_SH; this is in accordance with previous quantum chemical parameters, as indicated in Table 1. As we know, C_18_SH can be dissociated into C_18_S (after losing one H atom) on the substrate surface. Therefore, the adsorption of C_18_S is also considered in this paper [24]. As indicated in Figure 3, the adsorption energy per unit area of C_18_S decreases from −3.24 eV·Å^−2^ to −3.37 eV·Å^−2^ in solvents with an increased dielectric constant of 1 to 78.54. Therefore, the adsorption energy per unit area for C_18_S is much smaller than that for C_18_SH; this applies to the different solvent molecules as well. This indicates that C_18_S can be easily self-assembled on the Cu surface through competitive adsorption. Furthermore, the adsorption energy of C_18_S decreases in solvents with increased dielectric constant, which can well explain the previous experimental phenomenon that the C_18_S coverage on the Cu surface in the CH_3_CH_2_OH solution was higher than in the CH_3_(CH_2_)_4_CH_3_ solution at the assembly stage [13], as indicated in Figure S1.

### 2.3. The Interaction between C18SH and Solvent Molecules

**Figure 4:**
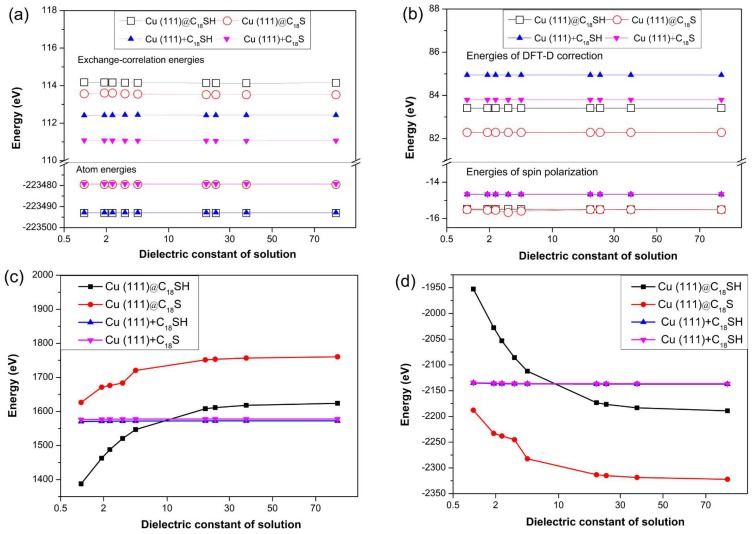
The energies of Cu (111) adsorption of C_18_SH in different solvents: (**a**) Atom and exchange-correlation energies; (**b**) spin-polarization and Density Functional Theory Dispersion (DFT-D) correction energies; (**c**) electrostatic energies; (**d**) vinetic energies. The @ sign stands for the adsorption state on the facet and the + sign stands for the sum of the separate energy. The x-axis labelled for dielectric constant of 1 Vacuum; 1.89–CH_3_(CH_2_)_4_CH_3_; 2.40–C_6_H_5_CH_3_; 3.42–C_2_HCl_3_; 4.81–CH_3_Cl_3_; 20.7–CH_3_COCH_3_; 24.3–CH_3_CH_2_OH; 37.5–CH_3_CN; 78.5–H_2_O.

In order to understand the reason for the adsorption energy changes in different solvents, we compare the different energies, as shown in Figure 4.

Based on Figure 4, the atomic energy, exchange-correlation energy, spin polarization and DFT-D correction energy are not greatly changed in different solvents. The electrostatic energies gradually increase while the kinetic energies decrease with increasing dielectric constant. Furthermore, the energies are not greatly changed if we sum the corresponding energy before adsorption, illustrating that the change is caused by the interaction between Cu (111) and C_18_SH (or C_18_S).

As we know, the C_18_SH is inherently non-polar and predominantly hydrophobic in nature, although the SH group provides a weakly polar character. When the C_18_SH solute molecule is surrounded by solvent molecules with different dielectric constants, the C_18_SH can generate a strong pull force with polar molecules and a weak compressive force with polar solvents. Although the increased polarizability of S compared to C provides a subtly greater polar character, SH groups are far less polarized than OH groups. Thus, a weak push force is generated in non-polar solvents, while a strong pull force is generated in non-polar solvents. When the C_18_SH is absorbed on the Cu (111) surface, the electrostatic interactions on the sulfur end disappears, but the electrostatic interactions on the other position still exist. As a result, the electrostatic interaction is quickly reduced in a polar solution (higher electrostatic energy means low interaction). The force on the hydrophobic end still exists after the adsorption, and it generates both a pull force in the non-polar solvent, and a compressive force in the polar solvent. The generated push force in the polar solvent limits the mobility of C_18_SH. Therefore, the kinetic energies for the adsorption of C_18_SH on Cu (111) decrease with increasing dielectric constant (more polar molecules).

**Figure 5:**
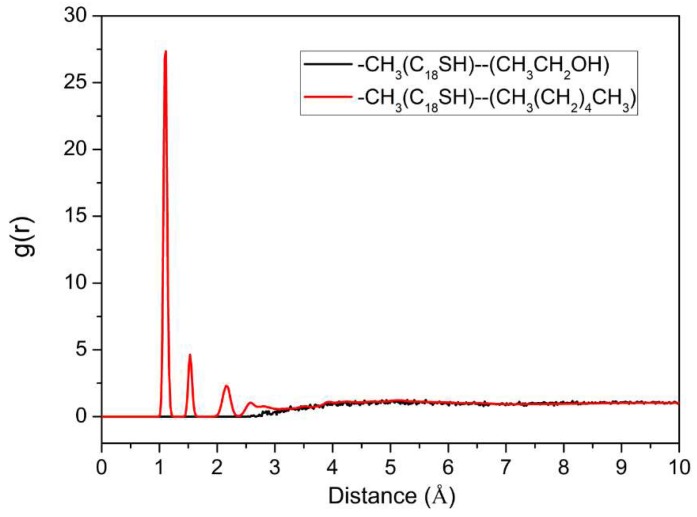
The radial distribution between the –CH_3_ site of C_18_SH and solvent molecules in a 24.3–CH_3_CH_2_OH and 1.89–CH_3_(CH_2_)_4_CH_3_ solution.

In order to understand the interaction between C_18_SH and solvent molecules, the radial distribution function between C_18_SH and the solvent was analyzed based on a molecular dynamics (MD) simulation, as shown in Figure 5. There is a strong peak at 1.2 Å in the CH_3_(CH_2_)_4_CH_3_ solution, and no obvious peak in CH_3_CH_2_OH solution. This indicates that there is a high probability for CH_3_(CH_2_)_4_CH_3_ to be distributed on the –CH_3_ site. This confirms that the –CH_3_ site has indeed a great interaction with CH_3_(CH_2_)_4_CH_3_ molecules and a weak interaction with CH_3_CH_2_OH molecules.

## 3. The Effect of Solvent Polarity on the Quality of Self-Assembled C_18_SH

**Figure 6:**
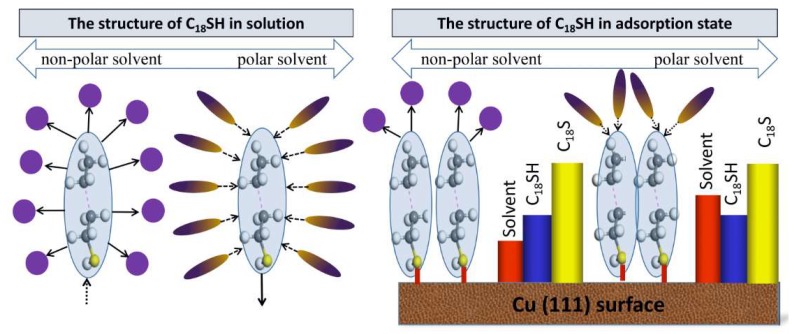
The adsorption of C_18_SH on the Cu (111) surface in polar and non-polar solvents, where the columnar chart represents the adsorption energies per unit area. The inward arrows present a push force, the outward arrows present a pull force, the solid line stands for a strong interaction, and the dash lines stand for a weak interaction.

Based on our previous results, the effect of solvent polarity on the quality of self-assembled C_18_SH on a Cu (111) plane can be illustrated in Figure 6.

As shown, the C_18_SH is embedded in the solute molecules, forming a cavity within the dielectric layer. The polarization charge distribution is determined by the generation of the charges on the cavity surface. The polarity can generate interaction forces between the molecules of solvent and the solute. Because of the predominantly hydrophobic property of C_18_SH in nature, C_18_SH generates a strong pull force with non-polar molecules and a weak compressive force with polar solvents. The solvent polarity effect is manifested through the interaction between SAMs and solvent molecules. When the C_18_SH adsorbs on the Cu (111) surface, the interaction between the –SH group and the solute disappears due to an intense chemical adsorption, while interactions between other positions of the C_18_SH and the solute still exists. The hydrophobic end mainly consists of CH groups, and it generates a great pull interaction with non-polar solvents. The generated pull interaction makes the adsorption unstable. Therefore, the kinetic energies of Cu (111) adsorbed by C_18_SH decrease along with increasing dielectric constant. A further MD simulation confirm that the –CH_3_ site does indeed have a great interaction with CH_3_(CH_2_)_4_CH_3_ molecules and a weak interaction with CH_3_CH_2_OH molecules. The above effect significantly increases the surface coverage of C_18_SH on Cu in an ethanol solution, compared with that in an n-hexane solution at the late stabilized stage.

## 4. Materials and Methods

Quantum chemical calculations can provide insights into the design of inhibitor systems with superior properties and elucidate the adsorption process at a molecular level [25]. The Dmol^3+^ module of the Materials Studio software (Accelrys Inc., San Diego, CA, USA) was employed for the quantum chemistry calculations. For the calculations, the Cu (111) facet has been widely chosen as an ideal model system to investigate the structure, stability, and adsorption properties, since it is the most stable surface under realistic conditions [26]. A supercell (4 × 4) was built with the following dimensions: 7.75 × 7.75 × 6.32 Å^3^ (as indicated in Figure S2). The Cu (111) lattice structure was divided into four layers, and the two bottom layers were constrained. The top site (above the Cu atom of the central plane), the bridge site (between the two Cu atoms, above the contact location), the face centered cubic (fcc) site (above the triangle of the Cu atoms on the plane, or directly above a Cu atom in the next layer below the plane), and the hexagonal close packing (hcp) site (above the triangle of Cu atoms on the plane, or directly above a Cu atom in the third layer below the plane) were considered during the adsorption study. The most stable adsorption site was determined based on the minimum energy of the system.

During the calculations, the self-consistent periodic Density Functional Theory (DFT) was used to study the relative stability and reactivity of the surface species on the Cu (111) surface. The Gradient-Corrected Functionals (GGA), in the form of the Perdew-Burke-Ernzerhof (PBE) approximation to the exchange-correlation energy, and the double-numerical quality basis, which was set with Double Numerical plus Polarization (DNP) functions, were employed. The Effective Core Potential (ECP) was used to handle the core electrons of the metallic atoms. Standard Kohn-Sham Density Functional Theory Dispersion (DFT-D) correction was used for the corrective calculation of van der Waals dispersion. A thermal smearing was adopted at 0.002 hartree, with a real-space cutoff at 4.4 Å. The k-point separation was at 0.04 Å^−1^. The solvent effect was considered by using a conductor-like screening model (COSMO) with different dielectric constant values [27]. The dielectric constants of water (H_2_O), acetonitrile (CH_3_CN), ethanol (CH_3_CH_2_OH), acetone (CH_3_COCH_3_), chloroform (CH_3_Cl_3_), trichloroethylene (C_2_HCl_3_), toluene (C_6_H_5_CH_3_), n-hexane (CH_3_(CH_2_)_4_CH_3_) and vacuum are 78.5, 37.5, 24.3, 20.7, 4.81, 3.42, 2.40, 1.89 and 1, respectively. The calculated lattice constants of Cu was consisted with the experimental result, as indicated in Table S2.

A Molecular Dynamics (MD) simulation was carried out with 1 C_18_SH and 100 solvent molecules in an amorphous cell. A COMPASS force field was used during the optimization as indicated in Figure S3. The initial models with 3D periodic boundary conditions were optimized via the smart minimizing method until the energy gradient reaches less than 0.1 kcal·mol^−1^. Moreover, the operating temperature was set at 298 K and controlled by means of the Nose thermostat method to match the real experiment procedure. Then, NVT (constant molecule numbers, volume and temperature) were used for the dynamic calculation. The van der Waals interaction was calculated using an atom-based method with a cutoff radius of 18.5 Å, while the long-range corrections were adopted outside 15 Å. The electrostatic summation method was calculated using the Ewald method with an accuracy of 10^−5^ kcal·mol^−1^ for the computation of long-range non-bond energies in periodic systems. The details of the coordinates of atoms before C_18_SH was adsorbed and after C_18_SH was adsorbed on the Cu surface in vacuum are indicated in Tables S3 and S4, respectively.

Some parameters of molecular orbitals, such as absolute electronegativity (*χ*), the global hardness (*η*), and the fraction of electron transfer (Δ*N*), were also calculated using Equations (1)–(3).

(1) $$\chi =\left(-E_{H}-E_{L}\right)/2$$

(2) $$\eta =\left(-E_{H}+E_{L}\right)/2$$

(3) $$\Delta N=\frac{\chi_{Cu}-\chi_{mol}}{2(\eta_{Cu}+\eta_{mol})}$$

where *E*_H_ and *E*_L_ are, respectively, the energies of the HOMO and the LUMO for the corresponding molecules. In order to calculate the fraction of electron transfer, a theoretical value for the absolute electronegativity of Cu is taken as 4.48 eV, and a global hardness as 0 eV·mol^−1^, assuming the Cu atoms are softer than the neutral metallic atoms [28,29].

The interaction energy *E*_ads_ between the Cu (111) surface and the molecules is computed by:

*E_ads_* = *E_total_* − *E_molecule_* − *E_surface_* (4)

where *E*_total_ is the total energy of the system in different solutions, including the energy of water molecules and the metal plane; *E*_molecule_ is the molecules energy in different solutions; *E_surface_* is the energy of the metal surface in different solutions. In the current definition, the higher negative value of *E*_ads_ indicates a more stable adsorption on the surface [30,31].

$E_{atom}\;$(atomic energies) is obtained from atomic reference data for electronic structure calculations. The $E_{elst}\;$(Electrostatic energy), *E_kine_* (kinetic energy), $E_{XC}\;$ (exchange-correlation energy), $E_{spin}$ (Spin-polarization energy), and *E_DFT_*_−_ (DFT-D correction energy) can be calculated by Equations (5)–(9), respectively [32].

(5) $$E_{\mathrm{elst}}=-0.5<Z\left|D\right|Z>-<\rho \left|D\right|Z>-<\rho \left|D\right|\tilde{\rho }>+0.5<\tilde{\rho }\left|D\right|\tilde{\rho }>$$

(6) $$E_{\mathrm{kine}}=0.5N_{f}k_{B}T$$

(7) $$E_{XC}\left[n\right]=\int^{\text{​}}n\left(r\right)\varepsilon_{XC}\left[n\left(r\right)\right]dr$$

(8) $$E_{\mathrm{spin}}\;=\;E\left(M_{0}\right)-E\left(0\right)$$

(9) $$E_{DFT-D}=S_{i}\overset{N}{\underset{i=1}{\sum }}\overset{N}{\underset{j>i}{\sum }}f\left(S_{R}R_{ij}^{0},R_{ij}\right)C_{6,ij}R_{ij}^{-6}$$

In the above equations, Z is the nuclear charges, D = BA^−1^B, B and A are Coulomb matrices, ρ is the electron density, $\tilde{\rho }\;$ is the auxiliary density to solve the Poisson equation for the electrostatic potential of the solute, and $N_{f}$ is the number of degrees of freedom. *k*_B_ is the Boltzmann constant, T is the absolute temperature, n(**r**) is the number of the particles, $\varepsilon_{XC}\left[n\left(r\right)\right]$ is the exchange-correlation energy per particle in a uniform electron gas, $E\left(M_{0}\right)$ is the energy for the ground-state magnetic moment in the absence of an external field, and E(0)  is the energy when the ground-state magnetic moment is equal to zero. *S*_i_ is the XC-functional dependent factor where *S_R_* ≠ 1 and S_6_ ≡ 1, *f*(*S_R_*$R_{ij}^{0}$, *R_ij_*)$f\left(S_{R}R_{ij}^{0},{\;R}_{\mathrm{ij}}\right)$ is the damping function to express short range $S_{R}R_{ij}^{0}$ by long–range *R_ij_*. $C_{6,ij}R_{ij}^{-6}$ is a long range isotropic potential.

## 5. Conclusions

Based on the previous experimental findings, solvent polarity plays an important role in the adsorption of C_18_SH SAMs on Cu surfaces. The effect of solvent polarity on the quality of self-assembled C_18_SH on Cu (111) surfaces has been systematically analyzed using first-principles calculations. The results have revealed the molecular mechanisms behind the effect of solvent polarity.

C_18_SH is inherently non-polar and predominantly hydrophobic in nature, although the SH group provides a weakly polar character. In a non-polar molecule solution, there is a great pull interaction between C_18_SH and non-polar molecules. However, in a polar molecule solution, there is a weak interaction between C_18_SH and polar molecules. Due to the great interaction between C_18_S and the Cu surface, C_18_SH can self-assemble on the Cu surface (*E*_ads_ = −3.37 eV and Δ*N* = 312.7). After this, the electrostatic interactions on the sulfur end disappears, but the electrostatic interactions on the other positions still exist. The adsorption energy decreases greatly with increasing dielectric constant (or polarity). This is mainly caused by the change of electrostatic energies and kinetic energies in different solutions, attributable to the different types of interaction. The electrostatic interaction is quickly reduced in polar solutions due to the disappearance of electrostatic interactions on the sulfur end, and the reduced kinetic energies are due to the solvophobic cage-type effects which limit the mobility of C_18_SH. A further MD simulation also verifies that the –CH_3_ site has indeed a strong interaction with CH_3_(CH_2_)_4_CH_3_ molecules and a weak interaction with CH_3_CH_2_OH molecules. This study helps us to better understand the micro-mechanisms of solvent polarity effects for C_18_SH adsorption on Cu surfaces.

## Figures and Tables

**Table 1:** Quantum chemical parameters derived for different molecules at 298 K. The absolute electronegativity (*χ*), the global hardness (*η*), and the fraction of electron transfer (Δ*N*), were calculated by Equations (1)–(3), as described in the Materials and Methods section.

Name	Species	*E_H_* (eV)	*E_L_* (eV)	*χ*	*η*	Δ*N*
Water	H_2_O	−0.255	0.054	0.100	0.155	14.0
Acetonitrile	CH_3_CN	−0.299	−0.012	0.156	0.143	15.1
Ethanol	CH_3_CH_2_OH	−0.228	0.043	0.092	0.136	16.0
Acetone	CH_3_COCH_3_	−0.214	−0.064	0.139	0.075	29.4
Chloroform	CH_3_Cl_3_	−0.272	−0.078	0.175	0.097	22.6
Trichloroethylene	C_2_HCl_3_	−0.226	−0.066	0.146	0.080	27.4
Toluene	C_6_H_5_CH_3_	−0.217	−0.038	0.128	0.090	24.5
*n*-hexane	CH_3_(CH_2_)_4_ CH_3_	−0.270	0.059	0.106	0.164	13.1
*n*-octadecanethiol	C_18_SH	−0.206	−0.005	0.105	0.101	21.7
Dislocated state	C_18_S	−0.201	−0.186	0.194	0.007	312.7

## Supplementary Materials

The following is available online. Figure S1: Impedance plots of C18SH SAMs on Cu surfaces in different solvents in 0.1 mol·L^−1^ KCl as the supporting electrolyte. Figure S2: Top and side view of the Cu (111) surface. Figure S3: MD model in different solutions. Table S1: Stable adsorption energies of different species on the Cu (111) surface. Table S2: The calculated lattice constants of Cu, as compared with experimental results of reference. Table S3: The coordinates of atoms on a Cu (111) surface after the optimization in vacuum conditions. Table S4: The coordinates of atoms when C_18_SH is adsorbed on the top site of the Cu (111) surface after the optimization in vacuum conditions.
