# Supplementary material for: Theoretical Insights into the Solvent Polarity Effect on the Quality of Self-Assembled N-Octadecanethiol Monolayers on Cu (111) Surfaces
Source: Molecules. 2018 Mar 22;23(4):733. doi: 10.3390/molecules23040733 (PMC6017570; doi:10.3390/molecules23040733)
Supplement: Supplementary file 1 [file molecules-23-00733-s001.pdf]

Supporting Information

Theoretical insights into the solvent polarity effect on quality of self-assembled n-octadecanethiol monolayers on Cu (111) surface

Jun Hu [1,*], Shijun He [1], Yaozhong Zhang [3], Haixia Ma [1], Xiaoli Zhang [1] and Zhong Chen [2,*]

[1] School of Chemical Engineering, Northwest University, Xi’an 710069, Shaanxi, China; heshijun0717@163.com (S.H.); mahx@nwu.edu.cn (H.M.); xlzhang@nwu.edu.cn (X.Z.)

[2] School of Materials Science and engineering, Nanyang Technological University, 50 Nanyang Avenue, Singapore 639798, Singapore;

[3] State Key Laboratory of Eco-hydraulics in Northwest Arid Region of China, Xi'an University of Technology, Xi’an 710048, Shaanxi, China; zhangyz@xaut.edu.cn (Y.Z.)

* Correspondence: hujun32456@163.com (J.H.); ASZChen@ntu.edu.sg(C.Z.); Tel.: +65-6790-4256 (Z.C.)

Received: date; Accepted: date; Published: date

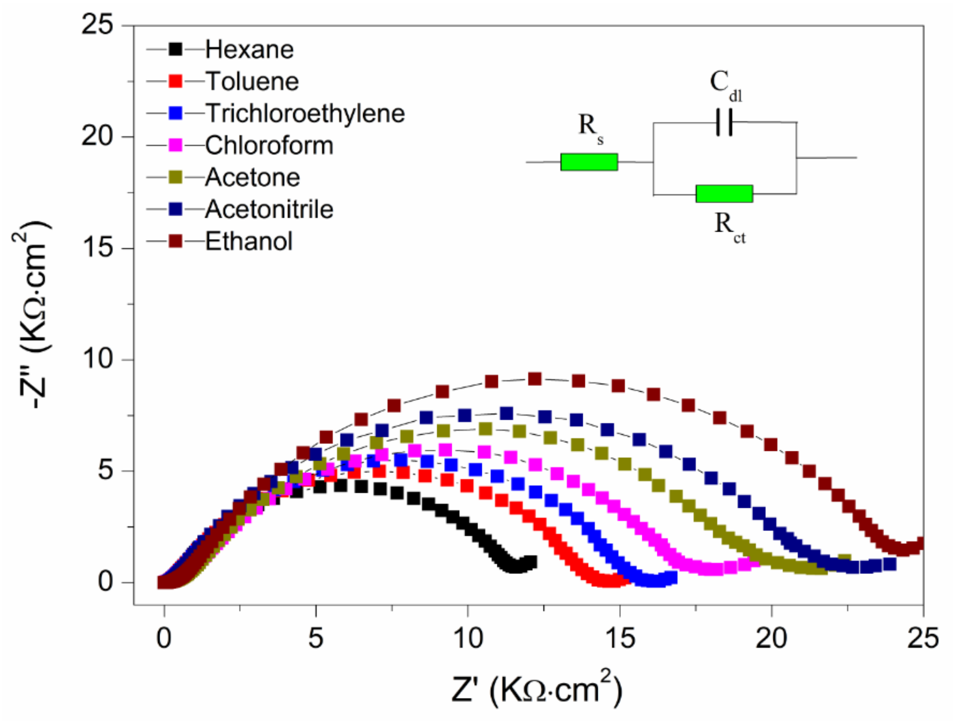

Figure S1. Impedance plots of $C_{18}SH$ (1 μmol Ⱡ$^{-1}$) SAMs on Cu surface in different solvents in 0.1 mol Ⱡ$^{-1}$ KCl as the supporting electrolyte for 24 h. The equivalent circuit is indicated as a solution resistance (R_0) in series with a parallel combination of interfacial capacitance (C_{dl}) and charge-transfer resistance (R_{ct}) The surface coverage θ=1-(R_0/R_{ct}), where R_0 is the charge-transfer resistance of bare Cu. The details assemble process was given as the following: Copper sample was mechanically polished by a semi-automatic polishing system (Tegramin-20, Struers, Denmark) using emery paste of 9, 3 and 1 μm (surface roughness of 1 ± 0.05 μm), then it was washed by deionized water and ethanol in the ultrasoniccleaner, respectively. The polished specimens were chemically etched in a mixed solution of H_2SO_4(3 M) and ethanol (3:1,v:v) to remove surface oxides. Then it was washed by deionized water and ethanol and immediately dried with a high-purity N_2. The pure copper samples were transferred in the airtight PMMA glovebox, which was filled with N_2, and were immersed in 1 μM solution of $C_{18}SH$ dissolved in various solvents for 24 h at the temperature of 25 °C. After that, all modified samples were rinsed by the corresponding pure solvents in the ultrasonically cleaner for 10 min to remove redundant thiol molecules and dried in N_2. Electrochemical impedance measurements of $C_{18}SH$-SAMs on copper samples were performed by an electrochemical work-station (Ametek, US) including a conventional three electrode system. The working electrode was the modified copper electrode (10 mm × 10 mm). The reference electrode and the auxiliary electrode were a saturated calomel electrode (SCE) and platinum foils, respectively. Test condition was an ac signal of 5 mV amplitudes using frequency range of 100 kHz to 0.1 Hz. Electrolyte solutions (0.1 M KCl) were deoxygenated by purified N_2. Film resistance and capacitance values were obtained by equivalent circuit fitting analysis using least square method.

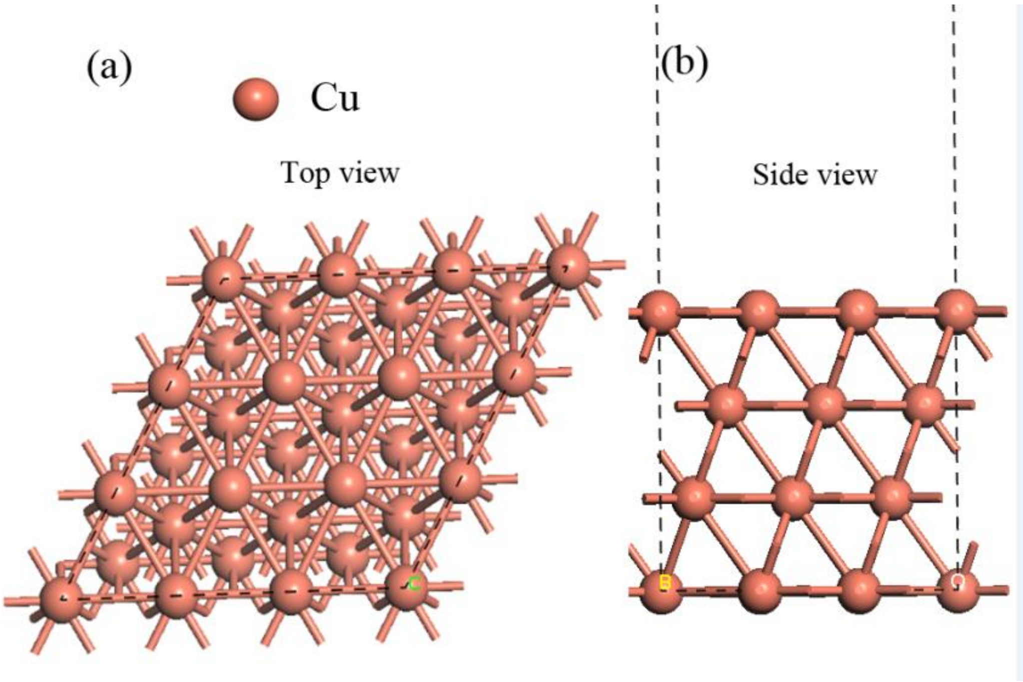

Figure S2. (a) Top view of the Cu (111) surface and (b) side view of a four-layer slab. The dash line defines the lattice of the slab model.

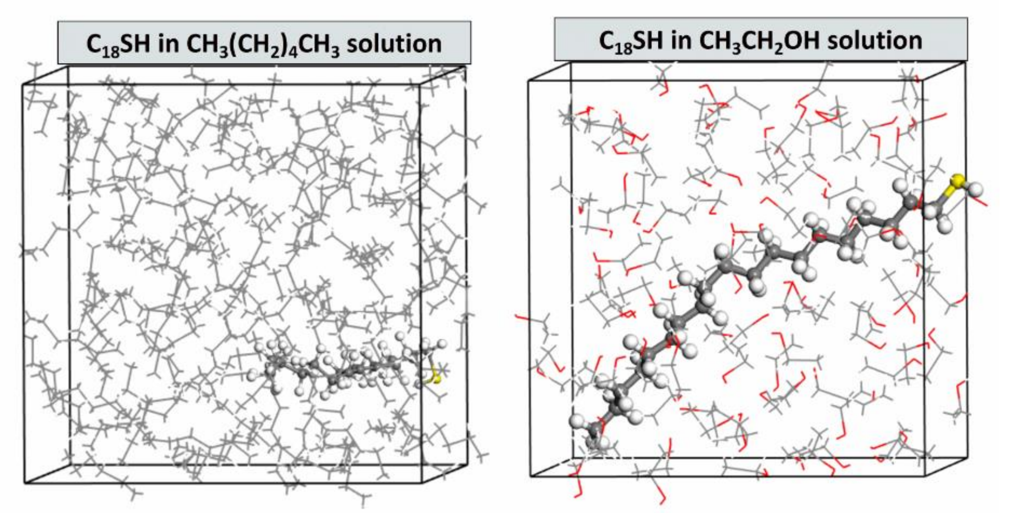

Figure S3. MD model in different solution. $C_{18}SH$ indicated as spheres and solvent indicated as line. Yellow spheres stand for S atoms, black spheres stand for C atoms, white spheres stand for H atoms, and red spheres stand for O atoms.

Table S1 Stable adsorption energies of different species on the Cu (111) surface.

Species	Solution	E_{total}	$E_{molecule}$	$E_{surface}$

		（Ha）	（Ha）	（Ha）
$C_{18}SH$	Vacuum	-8255.02019	-1145.41766	-7109.56205
$C_{18}SH$	$CH_3(CH_2)_4CH_3$	-8255.02311	-1145.42012	-7109.56205
$C_{18}SH$	$C_6H_5CH_3$	-8255.02410	-1145.42088	-7109.56205
$C_{18}SH$	C_2HCl_3	-8255.02545	-1145.42181	-7109.56205
$C_{18}SH$	CH_3Cl_3	-8255.02660	-1145.42254	-7109.56205
$C_{18}SH$	CH_3COCH_3	-8255.02992	-1145.42422	-7109.56206
$C_{18}SH$	CH_3CH_2OH	-8255.03014	-1145.42430	-7109.56206
$C_{18}SH$	CH_3CN	-8255.03065	-1145.42446	-7109.56206
$C_{18}SH$	H_2O	-8255.03126	-1145.42463	-7109.56205
$C_{18}S$	Vacuum	-8254.45606	-1144.77487	-7109.56205
$C_{18}S$	$CH_3(CH_2)_4$ CH_3	-8254.45867	-1144.77700	-7109.56205
$C_{18}S$	$C_6H_5CH_3$	-8254.45885	-1144.77768	-7109.56205
$C_{18}S$	C_2HCl_3	-8254.45885	-1144.77851	-7109.56205
$C_{18}S$	CH_3Cl_3	-8254.45920	-1144.77914	-7109.56205
$C_{18}S$	CH_3COCH_3	-8254.46052	-1144.78045	-7109.56206
$C_{18}S$	CH_3CH_2OH	-8254.46059	-1144.78059	-7109.56206
$C_{18}S$	CH_3CN	-8254.46074	-1144.78072	-7109.56206
$C_{18}S$	H_2O	-8254.46089	-1144.78079	-7109.56205
$CH_3(CH$	$CH_3(CH_2)_4$ CH_3	-7346.43827	-236.82299	-7109.56205
$C_6H_5CH_3$	$C_6H_5CH_3$	-7380.92244	-271.29977	-7109.56205
C_2HCl_3	C_2HCl_3	-8566.43287	-1456.83070	-7109.56205
CH_3Cl_3	CH_3Cl_3	-8528.37295	-1418.77607	-7109.56205
CH_3CO	CH_3COCH_3	-7302.58979	-193.00042	-7109.56206
CH_3CH_2	CH_3CH_2OH	-7264.51130	-154.91773	-7109.56206
CH_3CN	CH_3CN	-7242.23335	-132.64570	-7109.56206
H_2O	H_2O	-7185.97021	-76.38941	-7109.56205

Table S2 The calculated lattice constants of Cu, as compared with experimental results from reference [S1]. It indicates that the maximum deviation for optimized lattice constants is 0.011 Å, or 0.30 %, when compared with experimental value of 3.604 Å. The difference is well within the acceptable tolerance of 0.50 %, and hence the calculation method and model are satisfactory.

Source	a	b	c	α	β	γ
Our calculation	3.614 Å	3.615Å	3.614 Å	89.87 °	89.87 °	89.87 °
Experimental	3.604Å	3.604Å	3.604Å	90.00°	90.00°	90.00°
Relative error	0.28 %	0.30 %	0.28 %	0.14 %	0.14 %	0.14 %

Table S3 The coordinates of atoms on Cu(111) surface after optimization in vacuum condition.

	Atom	X	Y	Z
1	Cu	0.000	0.000	0.000
2	Cu	0.000	0.000	11.952
3	Cu	2.440	1.409	7.968
4	Cu	0.000	2.817	3.984
5	Cu	4.880	0.000	0.000
6	Cu	4.880	0.000	11.952
7	Cu	7.319	1.409	7.968
8	Cu	4.880	2.817	3.984
9	Cu	9.759	0.000	0.000
10	Cu	9.759	0.000	11.952
11	Cu	12.199	1.409	7.968
12	Cu	9.759	2.817	3.984
13	Cu	-2.440	4.226	0.000
14	Cu	-2.440	4.226	11.952
15	Cu	0.0000	5.634	7.968
16	Cu	-2.440	7.043	3.984
17	Cu	2.440	4.226	0.000
18	Cu	2.440	4.226	11.952
19	Cu	4.880	5.634	7.968
20	Cu	2.440	7.043	3.984
21	Cu	7.319	4.226	0.000
22	Cu	7.319	4.226	11.952
23	Cu	9.759	5.634	7.968
24	Cu	7.319	7.043	3.984
25	Cu	-4.880	8.452	0.0000
26	Cu	-4.880	8.452	11.952
27	Cu	-2.440	9.860	7.968
28	Cu	-4.880	11.269	3.984
29	Cu	0.000	8.452	0.000
30	Cu	0.000	8.452	11.952
31	Cu	2.440	9.860	7.968
32	Cu	0.000	11.269	3.984
33	Cu	4.880	8.452	0.000
34	Cu	4.880	8.452	11.952
35	Cu	7.319	9.860	7.968
36	Cu	4.880	11.269	3.984

Table S4 The coordinates of atoms when $C_{18}SH$ is adsorbed on the top site of Cu (111) surface after optimization in vacuum condition.

	Atom	X	Y	Z
1	Cu	0.000	0.000	0.000
2	Cu	-0.028	0.022	11.610
3	Cu	2.429	1.318	7.721
4	Cu	0.000	2.817	3.984
5	Cu	4.880	0.000	0.000
6	Cu	4.842	0.047	11.603
7	Cu	7.287	1.359	7.749
8	Cu	4.880	2.817	3.984
9	Cu	9.759	0.000	0.000
10	Cu	9.734	0.095	11.549
11	Cu	12.197	1.352	7.738
12	Cu	9.759	2.817	3.984
13	Cu	-2.440	4.226	0.000
14	Cu	-2.511	4.204	11.560
15	Cu	-0.027	5.610	7.781
16	Cu	-2.440	7.044	3.984
17	Cu	2.440	4.226	0.000
18	Cu	2.409	4.145	11.529
19	Cu	4.853	5.609	7.766
20	Cu	2.440	7.043	3.984
21	Cu	7.319	4.226	0.000
22	Cu	7.287	4.238	11.625
23	Cu	9.739	5.590	7.763
24	Cu	7.319	7.043	3.984
25	Cu	-4.880	8.452	0.000
26	Cu	-4.907	8.473	11.618
27	Cu	-2.414	9.796	7.785
28	Cu	-4.880	11.269	3.984
29	Cu	0.000	8.452	0.000
30	Cu	-0.106	8.462	11.692
31	Cu	2.421	9.810	7.788
32	Cu	0.000	11.269	3.984
33	Cu	4.880	8.452	0.000
34	Cu	4.952	8.457	11.617
35	Cu	7.292	9.808	7.763
36	Cu	4.880	11.269	3.984
37	C	2.045	9.930	18.394
38	C	0.916	11.299	20.685
39	C	2.421	10.670	23.083
40	C	1.168	11.378	25.593
41	C	2.703	10.423	27.860

42	C	1.377	10.596	30.428
43	C	2.904	9.312	32.529
44	C	1.588	9.169	35.105
45	C	3.109	7.614	37.021
46	C	1.894	7.308	39.639
47	C	1.745	9.740	41.207
48	C	0.859	9.289	43.925
49	C	0.791	11.679	45.559
50	C	0.111	11.160	48.327
51	C	0.068	13.530	49.991
52	C	-0.533	12.978	52.770
53	C	-0.573	15.335	54.453
54	C	-1.169	14.748	57.224
55	S	2.008	8.279	15.605
56	H	4.051	10.446	18.171
57	H	2.025	7.884	18.799
58	H	-1.072	10.711	20.931
59	H	0.880	13.359	20.361
60	H	4.306	11.558	22.964
61	H	2.773	8.612	23.104
62	H	-0.745	10.542	25.658
63	H	0.900	13.444	25.718
64	H	4.524	11.439	27.944
65	H	3.195	8.422	27.517
66	H	-0.497	9.685	30.289
67	H	1.014	12.590	30.923
68	H	4.738	10.287	32.749
69	H	3.371	7.370	31.918
70	H	-0.299	8.307	34.871
71	H	1.259	11.094	35.832
72	H	5.001	8.469	37.249
73	H	3.437	5.720	36.211
74	H	2.994	5.899	40.715
75	H	-0.018	6.493	39.427
76	H	0.478	11.118	40.288
77	H	3.631	10.635	41.244
78	H	2.120	7.887	44.822
79	H	-1.035	8.411	43.903
80	H	-0.568	13.043	44.751
81	H	2.656	12.615	45.479
82	H	1.482	9.796	49.117
83	H	-1.748	10.214	48.420
84	H	-1.331	14.887	49.238
85	H	1.919	14.490	49.866

86	H	0.871	11.624	53.519
87	H	-2.379	12.009	52.899
88	H	-1.977	16.691	53.709
89	H	1.274	16.298	54.325
90	H	-3.032	13.841	57.419
91	H	-1.184	16.471	58.387
92	H	0.243	13.453	58.039
93	H	1.294	6.066	16.696
94	H	0.000	0.000	0.000
95	H	-0.028	0.021	11.610
96	H	2.429	1.318	7.721
97	H	0.000	2.817	3.984

References

[S1] Fehrenbach, G.M.; Bross, H. Self-consistent spline augmented-plane-wave calculation: Ground-state properties of Cu. *Phys. Rev. B* **1993**, 48, 17703-17714.
